# Atg4 in autophagosome biogenesis

**DOI:** 10.18632/oncotarget.22714

**Published:** 2017-11-27

**Authors:** Jana Sánchez-Wandelmer, Fulvio Reggiori

**Affiliations:** Fulvio Reggiori: Department of Cell Biology, University of Groningen, University Medical Center Groningen, Groningen, The Netherlands

**Keywords:** autophagy, autophagosome, Atg1, Atg4, phosphorylation

Double-membrane autophagosomes are the hallmark of autophagy, a catabolic process conserved among eukaryotes. Autophagy is crucial for the degradation of unwanted structures such as unfolded proteins, protein aggregates and dysfunctional organelles, which, if accumulated, could impair cellular homeostasis. Hence, autophagy dysregulation leads to the development of several pathologies including neurodegeneration, metabolic diseases and cancer. Therefore, modulation of the autophagic flux is a new field being explored in the search of novel therapies for various illnesses. A better understanding on how autophagosomes are formed is thus crucial to find new molecular targets for drug development.

During the initial steps of autophagosome biogenesis, activation of the Atg1/ULK kinase triggers the recruitment of other autophagy-related (Atg) proteins to a specialized site known in yeast as the phagophore assembly site (PAS). At the PAS, a precursor cistern called phagophore is generated and subsequently elongated and sealed into an autophagosome. These principles are highly conserved and therefore also found in mammalian cells. The sequential association of the Atg proteins to the PAS and their cross-talk is tightly regulated in time and space to avoid the formation of aberrant and potentially cytotoxic intermediates. While Atg proteins decorate autophagosomal membranes throughout the expansion of phagophores, most of them are absent on complete autophagosomes prior to their fusion with vacuoles/lysosomes [1]. The disassembly of the Atg machinery from the surface of complete autophagosomes appears to be a prerequisite for this fusion event to happen [1,2]. The clearance of the phosphatidylinositol-3-phosphate (PtdIns3P) generated at the PAS during autophagosome biogenesis [1], from the outer membrane of autophagosomes allows the dissociation of PtdIns3P effectors such as Atg18 and the Atg12-Atg5-Atg16 complex. However, other mechanisms are required for the release of proteins that are covalently bound to membranes like Atg8. Atg8 is constitutively cleaved by Atg4 and upon autophagosome biogenesis induction, it is conjugated to the phosphatidylethanolamine (PE) present in autophagosomal membranes. The Atg8-PE conjugate mediates cargo selection and contributes to phagophore expansion and closure. Its deconjugation from autophagosomal membranes through the dissolution of the Atg8-PE bond, which is also mediated by Atg4, is equally important to ensure autophagy progression [2].

In our study, we proposed a model in which the Atg1 kinase can phosphorylate Atg4 at Ser307 on the surface of autophagosomes to locally inhibit Atg4 proteolytic activity [3]. This inhibition could contribute to the protection of the Atg8-PE pool required for autophagosome formation. Upon autophagosome completion, the inactivation of Atg1 and/or its dissociation will allow Atg4 to act on Atg8-PE and release Atg8 from its PE anchor, a step that could also trigger the disassembly of other Atg proteins from the surface of autophagosomes, promoting the subsequent fusion of the vesicles with vacuoles.

It is important to point out that Atg4 phosphorylation by Atg1 is very likely not the only way of regulating Atg4 proteolytic activity on autophagosomal membranes. For example, Atg4 is recruited onto autophagosomes by a recently described Atg8-PE association region (APEAR) motif, which preferentially binds Atg8-PE, and at least one C-terminal LC3-interacting region (LIR) motif [4]. These interactions are required for Atg8 deconjugation from PE [4]. A scenario where Atg4 is sitting on autophagosomal membranes and Atg1 is keeping it inactive can, however, be excluded. First, Atg4 appears to be recruited onto autophagosomes at a precise time point rather than being localized throughout their entire process of formation [4]. Second, Atg4 phosphorylation at Ser307 by Atg1 completely blocks its association with Atg8-PE and consequently the simultaneous binding to Atg8-PE through its APEAR and/or LIR motifs, and Atg1 phosphorylation are very unlikely [3].

Topologically, Ser307 is positioned at the entry of the groove accommodating the Atg4 catalytic site and therefore Atg1 cannot easily access it [3]. Interestingly, *Legionella* RavZ-mediated LC3-PE C-terminal processing relies on N- and C-terminal LIR motifs in the RavZ protease, which are involved in both the interaction with the substrate and the opening of the catalytic groove required to access the bond that has to be cleaved [5]. With this notion in mind, Atg1 could be part of a safeguard mechanism blocking a premature Atg8-PE deconjugation by Atg4. In particular, inappropriate association of Atg4 with its substrate on autophagosomal membranes and the opening of the catalytic groove to cleave Atg8-PE, would be blocked by Atg1 phosphorylation of Ser307 leading to the immediate dissociation of Atg4 from Atg8-PE (Figure 1). Upon autophagosome completion, recruitment of Atg4 and concomitant Atg1 inactivation, would allow Atg4 to cleave Atg8-PE (Figure 1). The apparent distribution of Atg1 over the entire surface of nascent autophagosomes is consistent with the notion of Atg1 being a guardian protecting the Atg8-PE pool on autophagosomal membranes [6]. However, it remains to be established whether Atg1 phosphorylates a pool of Atg4 present on autophagosomal membranes and/or those Atg4 molecules associating too early to the phagophore (Figure 1). Obviously, scenarios involving other regulatory mechanisms are also compatible with this model.

**Figure 1:**
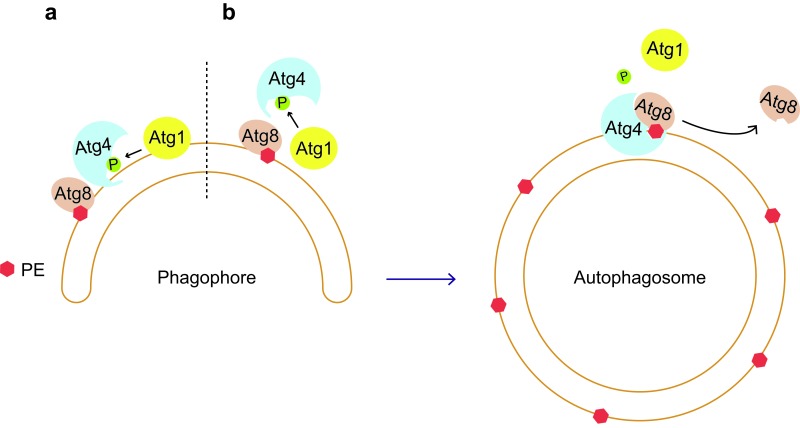
Model for Atg4-mediated regulation of Atg8-PE deconjugation on autophagosomal membranes Atg8 is conjugated to the PE in the membranes of the phagophore. Presence of active Atg1 at this location permits to phosphorylate Atg4 at Ser307, a modification that inhibits the interaction between Atg4 and Atg8-PE. As a result, Atg1 protects Atg8-PE from being prematurely cleaved, allowing the expansion of the phagophore into an autophagosome. Two scenerios can be envised, i.e. Atg1 phosphorylates (**a**) a pool of Atg4 already present on autophagosomal membranes or (**b**) Atg4 molecules that associates too early to the phagophore. Upon autophagosome completion, Atg1 gets inactivated and/or dissociates from membranes, allowing Atg4 to interact with Atg8-PE through its APEAR and C-terminal LIR motifs, which catalyzes the release of Atg8 from its PE anchor.

Remarkably, a similar inhibitory mechanism for the Atg4 deconjugating activity has been recently described in mammalian cells [7]. Although the Ser316 phosphorylated in ATG4B by ULK1, one of the mammalian homologues of Atg1 is conserved in Atg4 [7], it does not play a major role in the regulation of yeast autophagy [3]. Interestingly, the yeast Ser307 is conserved in only two of the four mammalian Atg4 isoforms, i.e. ATG4C and ATG4D [3]. These isoforms are less active than ATG4A and ATG4B, where there is an alanine at the equivalent position of Ser307. This suggests that Atg4 phosphorylation by Atg1 is a regulatory mechanism that, though evolutionary conserved, appears to have been redefined in organisms with higher complexity. This could be due to the necessity of a more accurate integration of metabolic, developmental and organismal signals modulating autophagy, but also of the involvement of Atg8/LC3 conjugation/deconjugation in other pathways such as LC3-associated phagocytosis, which very likely are regulated differently than autophagy.

## References

[1] Cebollero E Curr Biol.

[2] Yu ZQ (2012). Autophagy.

[3] Sánchez-Wandelmer J (2017). Nat Commun.

[4] Abreu S (2017). EMBO Rep.

[5] Kwon DH (2017). Biochem Biophys Res Commun.

[6] Suzuki K (2013). J Cell Sci.

[7] Pengo N (2017). Nat Commun.

